# Seroprevalence of *Toxoplasma gondii* infection in blood donors in mainland China: a systematic review and meta-analysis

**DOI:** 10.1051/parasite/2018037

**Published:** 2018-07-23

**Authors:** Taiwu Wang, Yifang Han, Zuanqin Pan, Hengzhong Wang, Meng Yuan, Hong Lin

**Affiliations:** 1 Research Institute for Medicine of Nanjing Command 293 Zhongshan Eastern Road Nanjing 210002 PR China; 2 Gaoyou Hospital Affiliated to Soochow University (Gaoyou People’s Hospital) Gaoyou 225600 PR China; 3 Faculty of Preventive Medicine, The Fourth Military Medical University 169 Changle West Road Xi’an 710032 PR China; 4 Jiangsu Province Blood Center 179 Longpan Road Nanjing 210042 Jiangsu PR China

**Keywords:** *Toxoplasma gondii*, Blood donors, China, Systematic review, Meta-analysis

## Abstract

*Toxoplasma gondii* transmitted from blood donors to receiving patients has become a concern as numerous articles about the epidemiology of *T. gondii* infection in blood donors from different provinces have been published in China. This study aimed to evaluate the seroprevalence of *T. gondii* infection in Chinese blood donors using a meta-analysis. A total of 40 eligible studies, published from 1986 to 2017 and covering 18 provinces and municipalities were included. Among a total of 49,784 Chinese blood donors, the overall IgG seroprevalence of *T. gondii* infection was 6.26% (95% CI: 4.62%–8.13%). The highest prevalence was in the Northeast of China and the lowest in Central China. The infection rate increased slowly over the years, but not significantly. A statistically significant correlation was found between the seroprevalence of *T. gondii* infection and the detection method and educational level (*p* < 0.01). There was no relationship between age, gender, occupation and blood type and seroprevalence of *T. gondii* (*p* > 0.05). The prevalence of antibodies to *T. gondii* in Chinese blood donors was lower than in other countries, but the risk of transfusion-transmitted toxoplasmosis still exits. More concise methods are still needed to evaluate the possibility of transfusion-transmitted toxoplasmosis from blood donors.

## Abstract (fr)

La transmission de *Toxoplasma gondii* des donneurs de sang aux patients receveurs est devenue un sujet de préoccupation car de nombreux articles sur l’épidémiologie de l’infection à *T. gondii* chez des donneurs de sang de différentes provinces ont été publiés en Chine. Cette étude visait à évaluer la séroprévalence de l’infection par *T. gondii* chez des donneurs de sang chinois à l’aide d’une méta-analyse. Un total de 40 études admissibles, publiées de 1986 à 2017 et couvrant 18 provinces et municipalités, ont été incluses. Sur un total de 49 784 donneurs de sang chinois, la séroprévalence globale des IgG de l’infection à *T. gondii* était de 6,26% (IC à 95%: 4,62%-8,13%). La prévalence la plus élevée était au nord-est de la Chine et la plus faible en Chine centrale. Le taux d’infection a augmenté lentement au cours des années, mais pas de manière significative. Une corrélation statistiquement significative a été trouvée entre la séroprévalence de l’infection par *T. gondii* et la méthode de détection et le niveau d’éducation (p < 0,01). Il n’y avait pas de relation entre l’âge, le sexe, la profession, le type sanguin et la séroprévalence de *T. gondii* (p > 0,05). La prévalence des anticorps contre *T. gondii* chez les donneurs de sang chinois est plus faible que dans les autres pays, mais le risque de toxoplasmose transmise par transfusion existe toujours. Des méthodes plus concises sont encore nécessaires pour évaluer la possibilité de toxoplasmose transmise par transfusion chez les donneurs de sang.

## Introduction

Toxoplasmosis, a worldwide disease in humans and most warm-blooded animals, is caused by the opportunistic protozoan *Toxoplasma gondii*. One-third of the world’s population is estimated to be infected by this parasite [25]. *T. gondii* infects humans mainly through oral, blood and congenital transmission [34]. *T. gondii* was discovered in 1908 [51] and first in 1955 in China, and the corresponding work was published in 1957 [51]. The first human case of toxoplasmosis in China was reported in 1964 [44]. Over the last few decades, epidemiological surveys have been conducted to monitor the prevalence of *T. gondii* in China. The prevalence was 5.17% (0.33% ~ 11.79%) in the first national investigation between 1988 and 1992 [52], and then rose to about 7.9% between 2001 and 2004 [56], and 12.3% between 2006 and 2008 [43]. As an opportunistic pathogen, *T. gondii* rarely causes serious symptoms in healthy humans. However, the prevalence of *T. gondii* infection is rising and the number of clinical cases in immunocompromised patients [57] is increasing, such as transplant recipients, HIV-positive individuals, and cancer patients, as well as patients with congenital toxoplasmosis and psychosis. More attention should be given to toxoplasmosis as a serious public health problem.

It has been confirmed that *T. gondii* is a transfusion-transmissible pathogen [29]. In a meta-analysis, the prevalence of *T. gondii* in blood donors was estimated to be 33% worldwide [8]. In China, researchers paid more attention to *T. gondii* during the 1980s–1990s than at present. *T. gondii* screening in blood donors was piloted in many provinces. Based on these screening data, *T. gondii* infection deferral was added to Blood Donor Healthy Check Guidelines in 2001 [22]. The rule stipulates that toxoplasmosis recovered blood donors should be deferred for six months. Although *T. gondii* infection rates in Chinese blood donors have increased, rates are still relatively low compared with other countries [8]. In recent years, knowledge of *T. gondii* was low, and raw meat consumption and exposure to domestic pets has increased. Most blood donors are surprised by questions on *T. gondii* in questionnaires, and ask what toxoplasmosis is. So far, a comprehensive study on the prevalence of *T. gondii* has not been performed. Therefore, we conducted a national systematic review and meta-analysis to assess the prevalence of antibodies to *T. gondii* in Chinese blood donors.

## Methods

This study is based on the preferred reporting items for systematic reviews and meta-analyses (PRISMA) checklist [23], used to search for and select studies, and to assess quality and extracted data. This was done by two researchers, independent of each other, to avoid bias.

### Search strategy

Firstly, an inductive electronic search using keywords for all potential articles was performed; secondly, a deductive approach was used by searching for and retrieving articles from reference sections of identified publications as well as review articles related to blood donors or donations in China. We searched for epidemiological studies that were conducted before December 2017 in five English-language databases, including PubMed, Springer Link, Science Direct, Web of Science, and Wiley Online Library, and three Chinese databases: Wanfang, China National Knowledge Infrastructure (CNKI), and VIP (WeiPu). The keywords used to search the databases were *Toxoplasma, Toxoplasma gondii, T. gondii, Toxoplasmosis, blood donation, blood donors, transfusion, Chinese and China*. To maximize outputs, each keyword was searched individually or in combination. Result agreement and discrepancies between results were examined by a third researcher.

### Inclusion and exclusion criteria

Selected manuscripts needed to fulfill the following inclusion criteria: (i) cross-sectional study; (ii) locations within mainland China; (iii) targeted objectives were blood donors; (iv) serological diagnostic methods of IgG were used; (v) exact total and positive numbers were provided; and (vi) a sample size greater than 100. Studies were excluded if they did not fulfill all these criteria.

### Data extraction

The desired data were recorded using a data extraction form which included title, year of publication, province, sample size, number of seropositive cases, and diagnostic methods by two reviewers (Wang and Lin) using the inclusion criteria. Data on risk factors such as gender, age, education level, occupation and blood groups were also extracted. Discrepancies were resolved by discussion between the two reviewers and by seeking the opinion of the third author (Pan), if necessary.

### Meta-analysis

To avoid the confidence interval (CI) being out of the 0–1 range, and to prevent a study from having a large weighting when the proportion becomes too small or too large [2], we calculated seroprevalence estimates with variance stabilizing double arcsine transformation [2]. In addition, if the prevalence obtained from studies was not normally distributed, the prevalence needed to be transformed.

Point estimates and their 95% confidence intervals (CIs) for the prevalence rate of antibodies to toxoplasmosis were calculated for each study. The random effects model was adopted for overall and subgroup analysis if obvious heterogeneity existed, otherwise the fixed effects model was used. Furthermore, both models were adopted to test the difference of the two models for sensitivity analysis. Statistical heterogeneity was evaluated by the Cochran Chi-squared test (with *p* < 0.10 indicating statistically significant heterogeneity) and the statistic *I* ^2^ [6] (heterogeneity with *I* ^2^ of 0%–40% was considered not important, while *I* ^2^ of 30 to 60% was moderate heterogeneity, *I* ^2^ of 50%–90% was substantial heterogeneity, and *I* ^2^ of 75%–100% was considerable heterogeneity). A forest plot was used to provide a comprehensive overview of the included studies according to research year. Potential sources of heterogeneity were investigated further by arranging groups of studies according to potentially relevant characteristics. In this study, subgroup analysis was stratified by detection methods, regions (Northwest, Southwest, Northeast, South China, Central China, East China and North China), age, gender, occupation, blood group, and education background. Furthermore, meta-regression was used to investigate any significant difference between/among subgroups and the value of IgG seroprevalence. The publication bias was examined by funnel plots. In addition, the statistical significance was assessed by Egger’s regression asymmetry test. For meta-analysis, we assumed that the included studies were a random sample from each study population. All analyses were carried out with R software version 3.4.1 (with the package “meta” [26] (version 4.8-4) for meta-analysis).

## Results

### Characteristics of the eligible studies

**Fig. 1.**
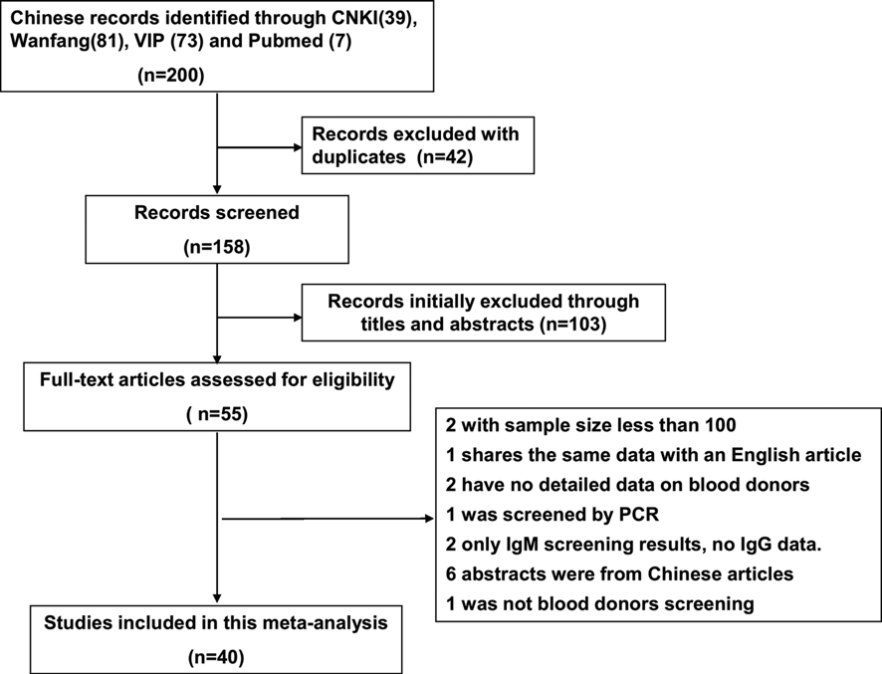
Flowchart describing the study design process.

Through our systematic review, a total should be 200 articles were found following the initial database search (Fig. 1). Table 1 shows the characteristics of the 40 studies [1, 3, 4, 7, 9–18, 21, 27, 28, 30-33, 35, 37–42, 45, 47–49, 53–55, 58–62] ultimately eligible for inclusion, which covered 18 provinces. The years in which the studies were performed and published ranged from 1985 to 2016 and from 1986 to 2017, respectively. The total number of blood donors was 49,784, with a range of 110–5068 per study, with one of two tests including Enzyme-Linked Immunosorbent Assay (ELISA, *n* = 31) [1, 4, 7, 10, 12, 13, 15–18, 21, 27, 28, 30–32, 35, 37–40, 42, 45, 47, 49, 53–55, 58, 60, 62], and the Indirect Hemagglutination Test (IHA, *n* = 9)[3, 9, 11, 14, 33, 41, 48, 59, 61] (Table1).

**Table 1.** Baseline characteristics of included studies based on geographical regions in China.

Region	Province	Author [reference]	Method	Publication year	Population	Number of IgG-positive sera	Prevalence (%)
Northwest	Shanxi	Ai et al. [16]	ELISA	2007	368	30	8.15
Northwest	Gansu	Wang et al. [17]	ELISA	1998	1480	150	10.14
Northwest	Xinjiang	Sun et al. [18]	IHA	1991	328	24	7.32
Southwest	Guizhou	Chen et al. [19]	ELISA	1999	500	32	6.40
Southwest	Guizhou	Hu et al. [20]	IHA	1991	200	1	0.50
Southwest	Sichuan	Wu et al. [21]	ELISA	1989	339	128	37.76
Southwest	Yunnan	Zhu et al. [22]	ELISA	2007	5068	1006	19.85
Southwest	Chongqing	Xu et al. [23]	ELISA	2017	1001	85	8.49
Northeast	Heilongjiang	Wang et al. [24]	ELISA	2002	264	56	21.21
North China	Hebei	Song et al. [25]	ELISA	2009	792	38	4.80
North China	Hebei	Song et al. [26]	ELISA	2012	1612	189	11.72
North China	Hebei	Wang et al. [27]	ELISA	2014	832	35	4.21
North China	Hebei	Yang et al. [28]	ELISA	2012	1056	51	4.83
North China	Hebei	Xin et al. [29]	ELISA	2013	864	44	5.09
North China	Hebei	Wu et al. [30]	ELISA	2017	1630	126	7.73
North China	Hebei	Shen et al. [31]	ELISA	2017	1165	83	7.12
Central China	Henan	Yang et al. [32]	IHA	1995	469	20	4.26
Central China	Henan	Luo et al. [33]	ELISA	2003	960	50	5.21
Central China	Henan	Sun et al. [34]	ELISA	2015	3200	98	3.06
Central China	Hubei	Gu et al. [35]	IHA	1989	2063	32	1.55
Central China	Hubei	Kuang et al. [36]	ELISA	2002	256	14	5.47
Central China	Hubei	Li et al. [37]	ELISA	2003	584	79	13.53
Central China	Hunan	Tong et al. [38]	ELISA	1994	1105	14	1.27
East China	Shandong	Feng et al. [39]	ELISA	1998	2025	259	12.79
East China	Jiangsu	Zhu et al. [40]	IHA	1987	300	17	5.67
East China	Jiangsu	Jiang et al. [41]	IHA	1991	212	12	5.66
East China	Jiangsu	Chen et al. [42]	IHA	1998	110	1	0.91
East China	Jiangsu	Wu et al. [43]	IHA	1994	1129	17	1.51
East China	Jiangsu	Zhu et al. [44]	IHA	1994	3542	156	4.40
East China	Jiangsu	Zhu et al. [45]	ELISA	1997	800	21	2.63
East China	Jiangsu	Yuan et al. [46]	ELISA	1998	723	15	2.07
East China	Jiangsu	Liu et al. [47]	ELISA	2001	2589	78	3.01
East China	Anhui	Wang et al. [48]	ELISA	1999	670	19	2.84
East China	Anhui	Shen et al. [49]	ELISA	2000	638	39	6.11
East China	Zhejiang	Meng et al. [50]	ELISA	1996	1197	215	17.96
East China	Zhejiang	Jiang et al. [51]	ELISA	2006	1023	58	5.67
South China	Guangdong	Zeng et al. [52]	ELISA	2005	680	49	7.21
South China	Guangdong	Zhong et al. [53]	ELISA	2010	1000	94	9.40
South China	Guangdong	Gu et al. [54]	ELISA	2010	4500	69	1.53
South China	Guangxi	Huang et al. [55]	ELISA	2013	2510	74	2.95

### Baseline characteristics of blood donors in included studies based on geographic regions

**Fig. 2.**
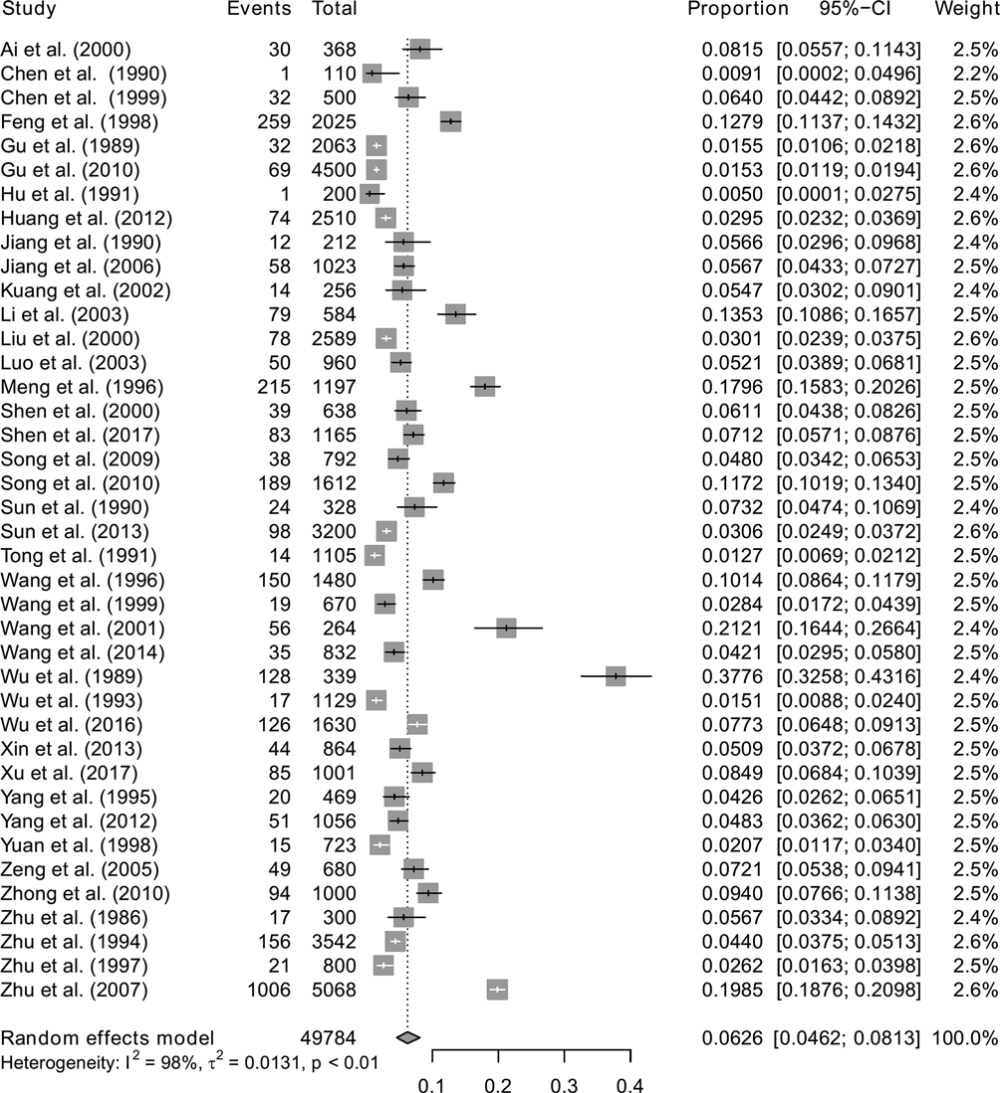
Forest plot of the overall seroprevalence estimates of *T. gondii* in blood donors.

A total of 3578 blood samples were found to have *T. gondii* IgG antibodies and the overall prevalence in blood donors was 6.26% (95% CI: 4.62%; 8.13%). The forest plot diagram of the current meta-analysis is presented in Fig. 2.

**Fig. 3.**
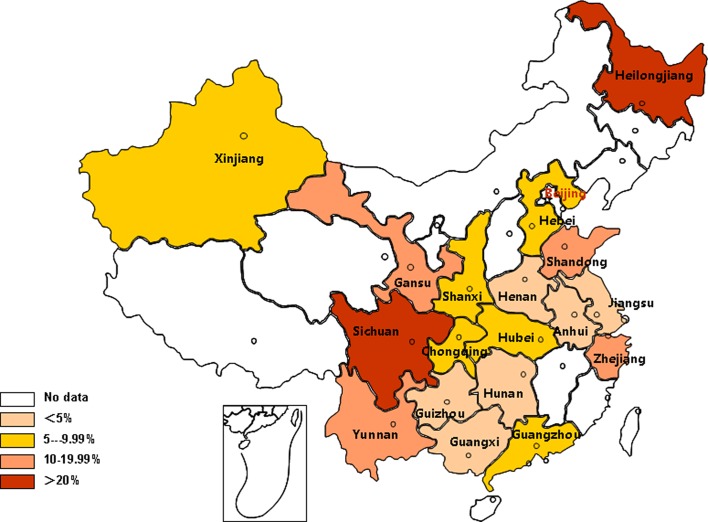
Geographic distribution of *T. gondii* prevalence.

Geographic analysis showed that the highest IgG prevalence of *T. gondii* infection was in Northeast blood donors (21.21%, 95% CI: 16.48%–26.36%) and the lowest in Central China (4.24%, 95% CI: 2.25%–6.82%) (Table 2). The prevalence rates of *T. gondii* in blood donors among different provinces are shown in Figure 3. The highest and lowest prevalence of *T. gondii* were found in Sichuan (37.76%, 95 %CI: 32.66%–42.99%) and Hunan (1.27%, 95 %CI: 0.68%–2.02%), respectively.

**Table 2.** Comparison of prevalence rates in different regions

				Heterogeneity test	
Regions	No. of studies	No. of donors	Prevalence [95% CI] (%)	*I* ^2^ (%)	*p*-value
Northwest	3	2176	8.95 [7.29; 10.76]	37.40	0.20
East China	13	14,958	4.85 [2.78; 7.43]	97.60	<0.01
Southwest	5	7108	11.93 [4.54; 22.16]	98.70	<0.01
Central China	7	8637	4.24 [2.25; 6.82]	96.00	<0.01
South China	4	8690	4.71 [1.88; 8.71]	98.00	<0.01
North China	7	7951	6.36 [4.55; 8.45]	92.20	<0.01
Northeast	1	264	21.21 [16.48; 26.36]		
Total	40	49,784	6.26 [4.62; 8.13]	98.00	<0.01

**Fig. 4.**
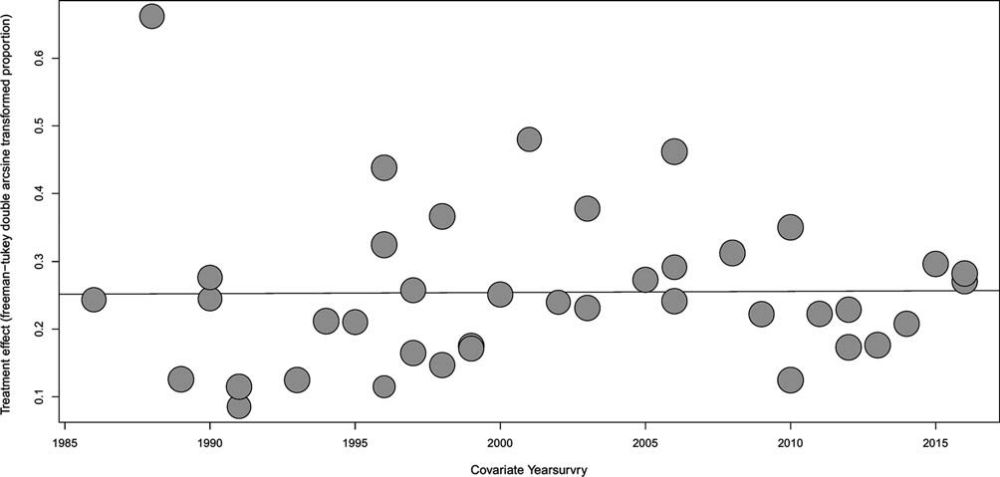
Meta-regression plot of antibodies to *T. gondii* according to the year of study. The overall prevalence of antibodies against *T. gondii* increased according to the year of study, but the trend was not significant (*p* > 0.05).

Concerning the meta-regression results for prevalence in different years, Figure 4 shows that there was no statistically significant difference in terms of the prevalence trends (*p* > 0.05). The lowest and highest prevalence was 1.07% (95% CI: 0.55%–1.73%) in 1991 and 21.21% (95% CI: 16.48%–26.36%) in 2001.

### Seroprevalence of *T. gondii* infection in blood donors in relation to risk factors

The pooled seroprevalence for each subgroup was calculated using the random-effects model, if there was high heterogeneity. The pooled estimates by potential various risk factors associated with *T. gondii* infection in blood donors are presented in Table 3. The seroprevalence of *T. gondii* tested with the ELISA method was 7.30% (95% CI: 5.25%–9.67%), and 3.16% (95% CI: 1.87%–4.76%) with the IHA method, and the difference between the two methods was significant (*p* = 0.002). The seroprevalence of *T. gondii* infection for different educational levels was significantly different (*p* = 0.006), with 4.80% (95% CI: 3.44%–6.37%) in the university-level population, 6.58% (95% CI: 4.79%–8.63%) in the high school population, and 9.01% (95% CI: 6.89%–11.38%) in the ≤ middle school population. There was no relationship between age, gender, occupation or blood type and seroprevalence of *T. gondii* (*p* > 0.05).

**Table 3.** Seroprevalence of *T. gondii* in blood donors associated with risk factors.

Factors	Categories	No. of studies	No. of blood donors	No. of IgG(+)	Prevalence [95% CI] (%)	Heterogeneity		Between-group differences*	
						*I* ^2^	*p*-value	*Q*	*p*-value
Method								9.46	0.0021
	ELISA	31	41431	3298	7.30 [5.25; 9.67]	98.70%	<0.01		
	IHA	9	8353	280	3.16 [1.87; 4.76]	90.40%	<0.01		
Age								0.99	0.6082
	18–30	15	15582	1471	7.37 [4.02; 11.61]	98.70%	<0.01		
	30–40	15	5458	469	8.49 [5.87; 11.53]	92.60%	<0.01		
	>40	15	2903	270	9.36 [6.10; 13.17]	88.70%	<0.01		
Gender								0.07	0.7983
	Male	22	16652	1369	6.75 [4.31; 9.67]	97.90%	<0.01		
	Female	22	14545	1104	6.21 [4.01; 8.83]	97.10%	<0.01		
Occupation								1.84	0.6061
	Students	10	5152	332	4.47 [1.83; 8.13]	96.30%	<0.01		
	Job-holders	10	6355	536	5.99 [2.57; 10.64]	97.50%	<0.01		
	Farmers	10	4611	561	8.49 [3.91; 14.54]	97.50%	<0.01		
	Others	8	2356	188	6.46 [3.22; 10.65]	92.00%	<0.01		
Blood								0.01	0.9997
	A	3	1421	104	7.74 [2.38; 15.69]	94.60%	<0.01		
	B	3	1773	140	7.36 [1.84; 15.99]	96.40%	<0.01		
	AB	3	406	28	6.97 [0.05; 21.35]	92.80%	<0.01		
	O	3	1382	95	7.23 [1.51; 16.50]	95.80%	<0.01		
Education								10.29	0.0058
	University	3	849	41	4.80 [3.44; 6.37]	0.00%	0.63		
	High school	3	2361	161	6.58 [4.79; 8.63]	72.00%	0.03		
	≤Middle school	3	641	58	9.01 [6.89; 11.38]	0.00%	0.72		

* Test for subgroup differences using random effects model.

Given the obvious difference between the two screening methods, we performed the analysis separately. Among the nine studies using the IHA method, sex data was provided in only three studies. We compared seroprevalence using different methods in men and women separately. In men, the seroprevalence using the ELISA method was 7.53% (4.81%; 10.78%), and using IHA 2.65% (0.52%; 6.23%). In women, the seroprevalence using the ELISA method was 6.78% (4.38%; 9.66%), and using IHA 2.11% (0.85%; 3.84%). The difference between the sexes was significant (*p* < 0.05).

### Publication bias and sensitivity tests

**Fig. 5.**
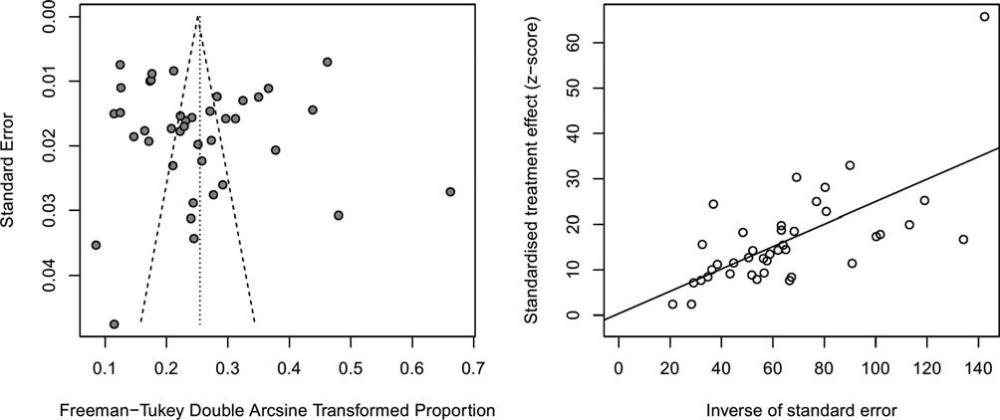
Funnel plot (left) and Egger’s publication bias plot (right), showing that no potential publication bias existed.

Funnel plot and Egger’s test were both used to examine publication bias. As shown in Figure 5, the funnel plot indicates no publication bias, which was also confirmed from Egger’s test (*t* = 0.11, *p* = 0.91). A sensitivity analysis was conducted for the pooled results by converting the pooled model (from the random effects model to the fixed effects model). The results demonstrated no large differences in proportions and 95% CIs before and after pooling, indicating stability in the pooled results.

## Discussion

Although *T. gondii* infection in China has been studied for 60 years [24] and many papers have investigated the prevalence of *T. gondii* in different populations, including blood donors, no systematic review on *T. gondii* in blood donors was carried out. In this study, we searched databases and identified a total of 40 relevant articles which contained eligible data on the seroprevalence of *T. gondii* infection in 49,784 blood donors across mainland China. To our knowledge, this is the first report to evaluate the national level of *T. gondii* seroprevalence in blood donors, which could be of great importance to public health surveillance and associated control policies.

The overall seroprevalence of *T. gondii* infection in blood donors in mainland China from 1986 to 2017 was 6.26% (95% CI: 4.62%–8.13%). Our study showed a low seroprevalence of *T. gondii* infection in blood donors in mainland China compared to the average seroprevalence of 33% (95% CI: 28%–39%) worldwide [8]. Compared to the prevalence recorded in other Asia countries, the prevalence of *T. gondii* in China was the lowest [8].

*T. gondii* is widely distributed, especially in warm, moist and low altitude regions, and at temperate to tropical temperatures oocysts remain infectious for up to 1.5 years [20]. In this study, it was interesting to note that the cold northeast regions of China, at high attitude, had the highest prevalence, and the warm and low attitude regions in the south of China had the lowest prevalence. The result was consistent with Pan’s review [24]. This may be related to economic development levels and sanitary conditions. Another reason was that the investigations available for the north of China were few; in some regions, only one province reported the prevalence of *T. gondii* infection.

In our research, there were two factors, namely screening methods and education levels, which were associated with *T. gondii* seroprevalence. An experiment that compared IHA, ELISA and another screening method with each other found that there was no significant difference in sensitivity and specificity between IHA and ELISA [50]. However, we found that testing methods may be one of the main sources of heterogeneity in this meta-analysis. In addition, it may be caused by the sample size and/or other confounding factors. Worldwide, *T. gondii* infection is associated with gender, age, contact with animals and raw meat consumption [8], but not with blood group. However, in Iran, the difference between men and women was not statistically significant [19]. In our study, lower educational level blood donors had higher seroprevalence, which may be related to the living conditions and chances of contact with animals.

There remain some limitations in our study. First, the articles in this current study were mostly published ten to twenty years ago; only six papers were published in the last five years. This shows that less attention has been focused on *T. gondii* infection in blood donors in recent years. Second, most of the studies focused on seroprevalence and the methods were varied; only two of them selectively detected DNA positivity for *T. gondii* [7, 59]. In addition, there was no investigation of the patients who received DNA-positive blood. Therefore, it could not be determined whether there was a possibility of transfusion-transmitted toxoplasmosis. Third, in terms of risk factors, only the characteristics of blood donors were analyzed in most of studies Risk factors like contact with animals (cats or dogs) or eating uncooked or raw meat and vegetables were not investigated in the articles. Fourth, to make the data comparable and to minimize the heterogeneity, we only adopted IgG as an indicator to analyze the prevalence of *T. gondii* infection. In diagnosis of *T. gondii* infection, the most useful indicator of active infection may be IgG and/or IgM. In this analysis of 40 studies, IgG and/or IgM was used in only 19 studies, IgM was used in two additional studies [5, 46]; the data about IgG and IgM need to be explored further.

In conclusion, the prevalence of antibodies to *T. gondii* in Chinese blood donors was lower than in other countries. However, the risk of transfusion-transmitted toxoplasmosis still exits. Only in one report, two patients were confirmed to have transfusion-transmitted toxoplasmosis from May 1986 to 1989 in China [36]. Although direct identification methods of *T. gondii* infection have been developed, routine use is not available in blood banks. In most Chinese blood centers, blood is processed by leukocyte filtration, which reduces the number of mandatory intracellular pathogens including *T. gondii*. The risk of *T. gondii* infection was also greatly reduced. Based on this, it is not necessary to screen *T. gondii* in blood donations in China.

## Conflict of interest

The authors declare that they have no conflict of interests.
